# The Epidemiology of Lower Limb Fractures: A Major United Kingdom (UK) Trauma Centre Study

**DOI:** 10.7759/cureus.56581

**Published:** 2024-03-20

**Authors:** James Zhang, Florence Bradshaw, Ishrat Hussain, Ioannis Karamatzanis, Michal Duchniewicz, Matija Krkovic

**Affiliations:** 1 School of Clinical Medicine, Department of Trauma and Orthopaedics, University of Cambridge, Addenbrooke's Hospital, Cambridge University Hospitals NHS Foundation Trust, Cambridge, GBR; 2 Department of Internal Medicine and General Surgery, Basildon University Hospital, Basildon, GBR; 3 Department of General Medicine, Basildon University Hospital, Basildon, GBR; 4 Department of Trauma and Orthopaedics, Addenbrooke's Hospital, Cambridge University Hospitals NHS Foundation Trust, Cambridge, GBR

**Keywords:** tibia, united kingdom, osteoporosis, fracture, epidemiology, femur, lower limb

## Abstract

Introduction: Understanding the epidemiology and incidence of fractures can help inform policymakers and clinicians about the needs of the population and highlight trends over time, allowing for tailoring of healthcare delivery to the population. This study reports on the lower limb fractures treated at a major trauma centre over a seven-year period.

Methods: We collected data on fracture locations, age, gender, BMI, hospital admission length, and treatment options of all lower limb fractures treated at a level I trauma centre from January 2015 to December 2021. We included data on the femur, tibia, and fibula, which were each split up into distinct regions. Fractures were subdivided by location and graphed, separated by gender, over age group. Finally, each location area’s frequency was graphed over the entire study period.

Results: A total of 8,511 patients sustained 8,613 fractures, given an overall incidence of 215.9 fractures per 100,000 patients per year. The mean age was 62.3 years, and 56.3% of patients were female. Fractures of the peri trochanteric region of the femur had the highest mean average age (79.9 years), which was closely followed by fractures of the head and neck of the femur (78.2 years). Fractures of the head and neck of the femur and the peri trochanteric region of the femur also had the highest proportion of females suffering from these fractures (67 and 66% female, respectively). Femur shaft fractures had the lowest average age (36.5 years) and the lowest proportion of female patients (29%). On graphing by location, separated by gender, over age group, overall fractures showed a bi-peak distribution of younger males and older, post-menopausal females having their respective peaks. Three further distinct distributions were observed in individual location fractures.

Conclusion:Identifying the relative incidence and demographic associations with lower limb fractures helps highlight a changing population’s needs. There is an absence of such study in literature in the United Kingdom (UK) since 2006. Our study’s insights and results aid clinicians and policymakers in the creation of guidelines and the distribution of resources based on the most recent information and elucidate changing healthcare service needs for the population.

## Introduction

There are very few studies providing a detailed analysis of the epidemiology of lower limb fractures. The last United Kingdom (UK)-based study commenting on the epidemiology of lower limb fractures was published in 2006 by Court-Brown et al., which focused on other fracture locations and the lower limb [1]. A previous study published in 2004 focused on the epidemiology of lower limb fractures [2]. These studies have advantages and disadvantages in commenting on the epidemiology of lower limb fractures in the UK. Court-Brown et al.'s paper focused on all fracture types but only covered one year, and data were only taken from an orthopaedic trauma unit in Edinburgh [1]. In addition to only providing a short snapshot in time, only focusing on the orthopedic trauma unit can provide biases in the data as not all the fractures that occurred in the population served by the hospital may be referred there. Despite the 2004 study covering 12 years and taking data from 142 general practices (GPs) across the UK, the data were obtained from GP records [2], which might have incomplete patient records, thus missing fractures.

Despite the lack of data in the UK, other papers have extensive data on the epidemiology of lower limb fractures in their respective countries. Hemmann et al. [3] in 2021 published a paper on the changing epidemiology of lower extremity fractures in adults over 15 years in Germany. Despite not considering fractures in patients under 15 years and using the International Classification of Diseases 10 (ICD-10) fracture coding, the study was national and covered an extended period. In addition, a similar study by Beerekamp et al. [4] in 2017 covered the epidemiology of extremity fractures in the Netherlands. This study covered a period of eight years and used national registries. However, similar to the German study, Beerekamp et al. disregarded those under 15 years, used ICD-10 coding, and divided the body into generalized body areas, for example, hip/upper leg and lower leg [4], losing some in-depth epidemiology analysis of fractures. Finally, a study by Wennergren et al. investigated the epidemiology and incidence of tibia fractures over five years at a hospital in Sweden. This paper again removed patients below the age of 15 but used the Arbeitsgemeinschaft für Osteosynthesefragen/Orthopedic Trauma Association (AO/OTA) classification system. Wennergren et al. also only focused on the tibia, missing out on other parts of the lower extremity [5]. Even though these studies have limitations and are international, they can still provide essential observations and comparisons for further epidemiology studies.

Court-Brown et al. found the overall incidence of fractures in men was 11.67/1000 people/years and 10.65/1000 people/years in women [1]. Meanwhile, Kaye et al. found a fracture incidence of 3.4/1000 people/years in women and 2.9/1000 people/years in men, showing wide variations in the reported incidence of fractures in the UK [2]. One of the reasons could be explained by the source of data collection, as discussed above. In addition, the defined population from which these incidences are calculated will further contribute to the variations in the reported fracture incidence.

Identifying and defining epidemiological trends in lower limb fractures enables policymakers and clinicians to make decisions about the distribution of healthcare services and delivery [6]. This is especially important in the UK due to the aging population. In 2011, 16.4% of the UK population was ≥ 65 years old, which rose to 18.6% in 2021. Using epidemiological data, we can identify which fractures could be pathological due to osteoporosis and aging [7]. As osteoporotic fractures account for approximately 2.4% of the healthcare budget in the UK [5], identifying these fractures could help set up preventative measures to reduce the frequency of these osteoporotic fractures and reduce the burden on the UK healthcare budget.

To further define the epidemiology of lower limb fractures, this study looked retrospectively at the lower limb fractures treated at a major trauma centre in the UK over seven years and aimed to provide a more in-depth locational analysis of fracture incidence in the lower limb.

## Materials and methods

Addenbrooke’s Hospital is the Major Trauma Centre in the Cambridgeshire region, UK, and the Care Quality Commission estimates it serves a population of approximately 570,000 patients [8].

The EPIC SystemsTM system was searched for all patients sustaining fractures to the long bones of the lower limb (femur, tibia, and fibula), who attended the Accident & Emergency Department or were referred directly to the Orthopaedics Outpatient Department, between 1 January 2015 and 31 December 2021. Inclusion was based on the relevant ICD-10 fracture coding applied to the patient at the point of admission being specific to one of the three included bones.

With each patient, the sub-location of the fracture was refined into the following subcategories: femur head or neck, femur peri trochanteric, femur subtrochanteric, femur shaft, distal femur, proximal tibia, tibial shaft, distal tibia (excluding medial malleolus), medial malleolus, lateral malleolus, and fibular shaft (excluding lateral malleolus fractures).

The patients’ demographic and presentation features of admission date, age, gender, days in the hospital, American Society of Anaesthesiologists (ASA) grade (for surgically treated patients), and body mass index (BMI) were also collected.

Using these data, the incidence of each fracture location per 100,000 patients per year was calculated. For demographic analysis, the mean of each variable (or proportion for gender) for each location was compared using a T-test with the entire cohort’s mean, with p < 0.05 being deemed statistically significant.

The overall cohort, as well as each individual fracture location, split the patients into five-year age intervals to show patterns and peaks of increased incidence. For this graphic, peritrochanteric and subtrochanteric fractures were combined into trochanteric region fractures, and distal tibia, medial, and lateral malleolus fractures were combined into ankle fractures.

Finally, the fracture incidence over the entirety of the inclusion period, split into intervals of each year and each quarter, was graphed for each location.

All data analysis and graph construction were performed via Statistical Product and Service Solutions (SPSS, v28; IBM SPSS Statistics for Windows, Armonk, NY) and Microsoft ExcelTM (Microsoft® Corp., Redmond, WA).

## Results

Overall, 8,511 patients suffered from 8,613 fractures in our studied time period. In the observed population, the average age was 62.3 years, and 56.3% were female. The overall incidence of lower limb fractures was 215.9 lower limb long bone fractures per 100,000 patients per year.

Location of lower limb fractures

Each sublocation’s overall incidence of fractures per 100,000 patients per year and the percentage of fractures that are open are shown in Table 1. The most common location of lower limb long bone fractures was the distal tibia and the femoral head and neck, making up 21.7% and 25.8% of total fractures, respectively. The subtrochanteric region of the femur and the medial malleolus were the two least common fracture locations, with each region representing under 3% of the total cohort.

**Table 1:** A breakdown of each location’s fracture frequency, percentage of total cohort, incidence per 100,000 patients per year, and percentage that are open fractures.

Location	Open vs Closed	Number	Location Percentage (%)	Location Incidence (/100,000 Patients per Year)	Percentage Open (%)
Femur head and neck	Closed	2210	25.81%	55.71	0.58%
	Open	13			
Femur peri-trochanteric region	Closed	1477	17.19%	37.12	0.27%
	Open	4			
Femur subtrochanteric region	Closed	144	1.70%	3.66	1.37%
	Open	2			
Femur shaft	Closed	376	5.36%	11.58	18.61%
	Open	86			
Femur distal	Closed	263	3.99%	8.62	23.55%
	Open	81			
Proximal tibia	Closed	675	8.67%	18.72	9.64%
	Open	72			
Tibial shaft	Closed	279	5.60%	12.08	42.12%
	Open	203			
Fibula	Closed	278	3.51%	7.57	7.95%
	Open	24			
Medial malleolus	Closed	200	2.75%	5.94	15.61%
	Open	37			
Lateral malleolus	Closed	303	3.73%	8.05	5.61%
	Open	18			
Distal tibia	Closed	1528	21.69%	46.82	18.20%
	Open	340			

The tibial shaft had the highest incidence of open fractures at 42.1%, with the femur shaft, distal femur, and distal tibial locations all representing around 20% of open fractures. The femur head and neck area and peri-trochanteric regions experienced the lowest incidence of open fractures at under 1% each.

Demographic breakdown of lower limb fractures

A further demographic breakdown of each fracture location and subtype is displayed in Table 2. The location with the most fractures was the head and neck of the femur, with 2,223 fractures. On the other hand, fractures of the peritrochanteric region of the femur had the lowest number recorded with 146. The highest mean average age (79.9 years old), which was closely followed by fractures of the head and neck of the femur (78.2 years old). On the other hand, fractures of the femoral shaft saw a considerably younger mean age of 36.5 years old.

**Table 2:** A demographic and admissions breakdown of each location of fracture.

Location	Number	Age Means (Years)	Proportion Female	ASA Mean	BMI Mean	Proportion Surgical Managed	Admission Length Mean (Days)
Femur head and neck	2223	78.16	0.67	2.73	24.06	0.9	15.61
Femur peritrochanteric	1481	79.89	0.66	2.86	23.94	0.86	17.05
Femur subtrochanteric	146	72.44	0.65	2.83	24.92	0.89	21.56
Femur shaft	462	36.53	0.29	2.43	24.56	0.92	18.82
Femur distal	344	53.87	0.48	2.66	25.19	0.67	20.73
Proximal tibia	747	50.90	0.51	2.31	25.21	0.49	14.15
Tibial shaft	482	39.61	0.32	2.30	24.83	0.66	15.12
Fibula	302	56.83	0.49	2.26	24.77	0.23	11.98
Medial malleolus	237	47.51	0.53	2.37	24.77	0.51	14.83
Lateral malleolus	321	53.59	0.46	2.21	25.57	0.36	9.89
Distal tibia	1868	49.36	0.54	2.31	24.79	0.63	10.58
OVERALL	8613	62.25	0.56	2.55	24.51	0.73	14.59

When looking at the gender breakdown of each fracture, fractures of the head and neck of the femur and the peritrochanteric region of the femur were associated with the highest proportion of females (67% and 66% female, respectively) and the greatest mean age. Further reflecting on our observation for the youngest mean age, femoral shaft fractures also had the lowest proportion of female fractures (29% female).

In our study, the average BMI was 24.5. There is relatively little variation in the mean BMI across fracture locations, with the range being between 23.9 for peri trochanteric fractures and 25.6 for the lateral malleolus.

ASA grade, surgical management, and lower limb fracture

In this study, 83.0% of fractures underwent surgical management. Table 2 provides further demographic information relating to ASA grade and surgical management. The ASA is used as a graded risk tool to predict the operative risk of patients based on the patient’s comorbidities [9]. Our study took the ASA grade of the patient at the initial operation and found that the mean ASA grade of those undergoing operation was 2.55. A higher ASA grade was associated with peri-trochanteric femur fractures, whereas lower ASA grades were associated with fractures of the lateral malleolus.

Age, gender, and lower limb fracture

**Figure 1:**
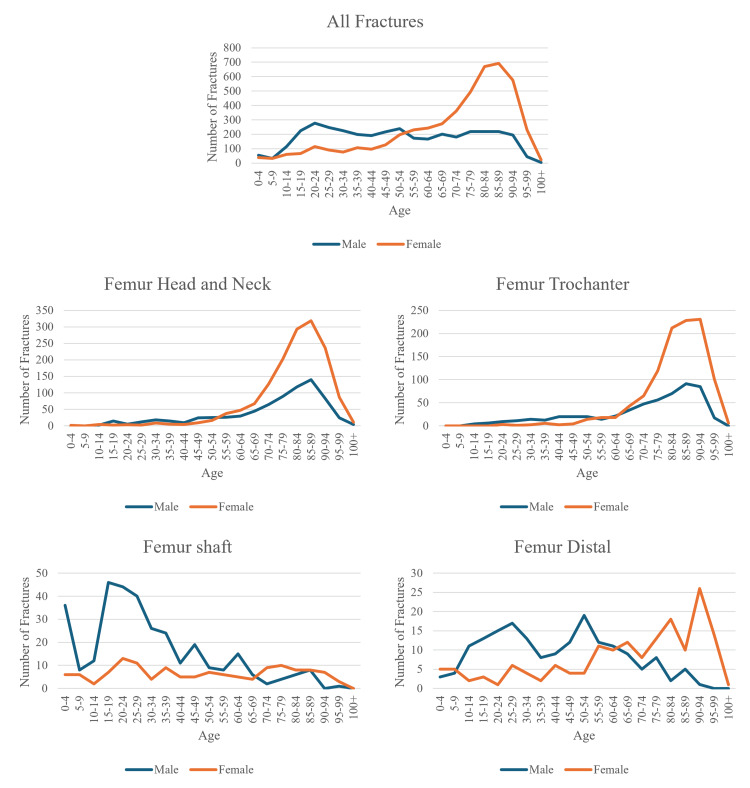
Femoral fracture location incidence by gender graphed with respect to age.

Each fracture location, alongside the entire cohort, was split into genders and had their total frequencies graphed with respect to age (Figure 1). In our study, subtrochanteric and peri-trochanteric fractures were subsequently grouped as “trochanter” fractures, and distal tibia, medial malleolus, and lateral malleolus were grouped as “ankle” fractures. Each location then had all fracture incidences graphed by age group in intervals of five years and separated by gender. This method of creating standard age and fracture incidence curves can help characterize epidemiology fracture patterns and was first done by Burh et al. in 1959 [10].

The distribution of overall lower limb fractures displays a bi-peak pattern, with younger males and older females having their respective peaks within each gender, with the female incidence beginning to increase in the fifth decade of life, during the menopausal period, with a large rate of rise, peaking at the ninth decade of life.

This same bi-peak pattern is shown in the ankle, fibula, and distal femur fractures as well, albeit with each of the locations having a more prominent younger male peak compared to the overall graph. In femur head and neck, and trochanteric fractures, the same pattern, without the younger male peak, is seen, with both genders having similar incidences until the sixth decade of life, after which female patient fractures rise rapidly. Tibial and femur shafts on the other hand lack the post-menopausal peak and only have a younger male peak around the third decade of life. Finally, proximal tibia fractures show a similar pattern for male and female patients, peaking in incidence in the sixth decade; however, at every stage, female patients have a higher incidence.

Fractures due to osteoporosis

**Figure 2:**
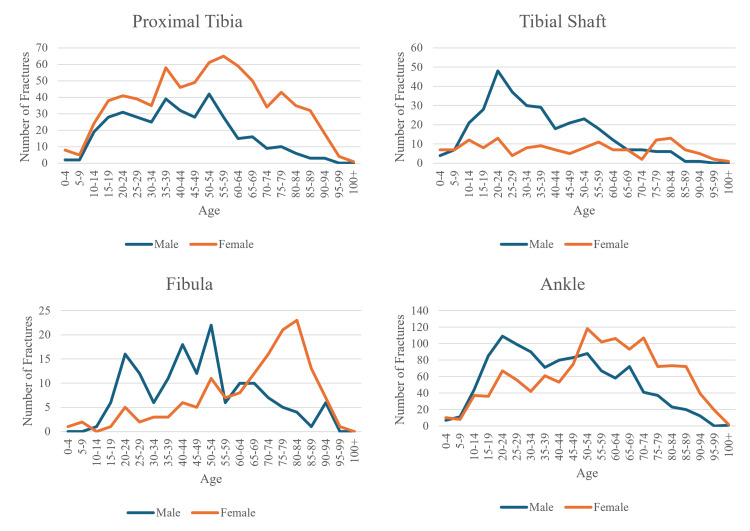
Lower leg fracture location incidence by gender graphed with respect to age.

Looking at Figures 1-2, it is likely that fractures of the femur head, neck, trochanter, distal femur, and fibula are osteoporotic due to the involvement of elderly women. This observation agrees that other femoral fractures and fibula fractures could be osteoporotic, but our data do not agree that tibia fractures are osteoporotic [1]. The shape of the graph for ankle fractures does not seem to suggest osteoporotic fractures; however, there has been a debate on whether ankle fractures are osteoporotic. We have grouped ankle fractures together, but due to the many different bones in the ankle, different types of ankle fractures will present in different groups of patients. It is suggested that bi-malleolar and tri-malleolar fractures are osteoporotic [7].

Lower limb fractures over time

**Figure 3:**
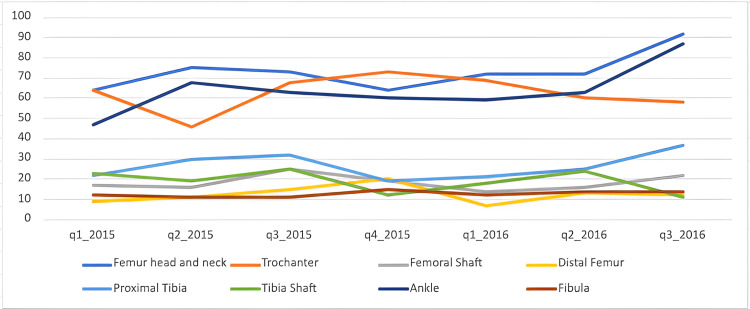
Fracture incidence over the study time period in years, by location of fracture.

**Figure 4:**
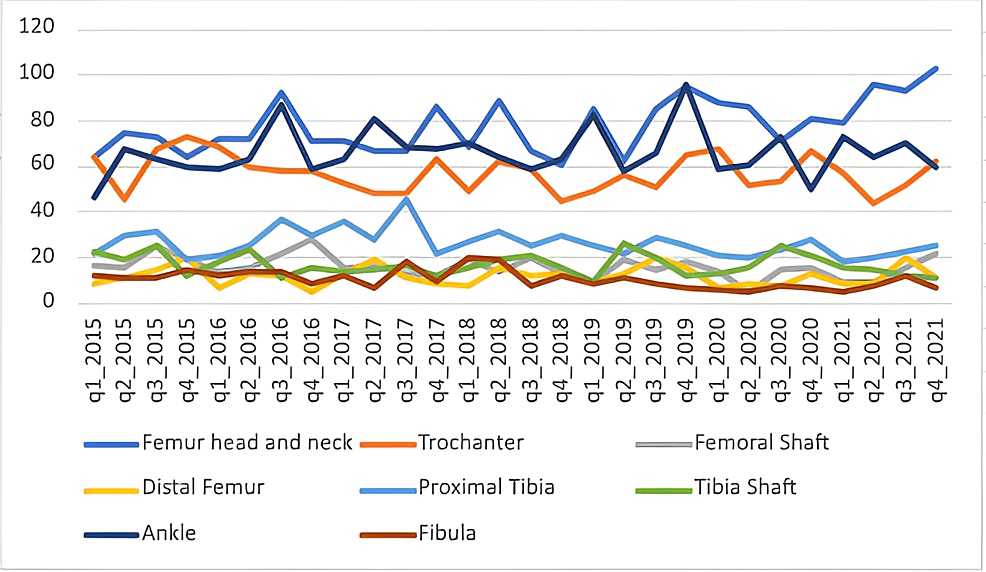
Fracture incidence over the study time period in quarters, by location of fracture.

Our study graphed the incidence of fractures overall and each fracture location over time, both by year and by quarter (Figures 3-4). Overall, our data observed a slight decrease in overall fractures in 2020, driven by a drop in quarter 2 (April until June inclusively). When each of the fracture locations is graphed separately, there is no dramatic drop in one specific fracture location in the second quarter of 2020.

When looking at the quarterly variation in fracture across a year, the number of fractures rises and falls across the year. Generally, there are increases in fracture numbers seen in the second and third quarters of each year. This variation in fracture incidence could be driven by warmer weather in the summer months, possibly leading to increased recreational activities [11].

## Discussion

The epidemiology of lower limb fractures is poorly described in the literature. In our study, the overall incidence of lower limb fractures was 215.9 lower limb long bone fractures per 100,000 patients per year. This value is lower than the incidence, which was quoted by other UK studies [1,2]. One of the main reasons for this could be that we are comparing incidence values that consider fracture locations other than the lower limb [1]. Furthermore, the defined population that the trauma center serves is uncertain. The possible reasons for this are the variable ambulance dispatch destination, hospital catchment variability, and patients selecting hospital services according to vicinity and personal preference, influencing the overall incidence of the fractures that we reported in this study. This uncertainty in the incidence estimate could be minimized by carrying out a whole nation study on lower limb fractures, similar to the method seen in the study in Germany [3]. However, carrying out and coordinating a national study collecting data across many trusts on lower limb fractures would be very challenging. One reason for this would be the heterogeneity of data recording by different trusts within the UK. However, a national study would have the advantage of revealing differences in service needs for fracture management across the UK.

Moreover, a factor that could have contributed to our study's lower reported fracture incidence was that we recorded bones with multiple fractures (comminuted fractures) as a single fracture. For example, if a patient had a comminuted proximal humerus fracture, our methods would consider this as a single proximal humeral fracture.

An important factor in this epidemiological study is the location of fractures. Our study's most common lower limb long bone fracture location was the distal tibia and the femoral head and neck. A large UK study in 2004 found the highest number of fractures at the ankle, followed by the tibia/fibula, the hip, and the tarsal/metatarsal bones [2]. There is a lack of newer UK studies, so we would ideally compare our quoted incidence value to a more recent similar study, such as that done by Hemmann et al. in Germany. Still, this paper does not mention an overall fracture incidence for the lower extremity [3]. As such, it is hard to compare our data against that of the most recent UK epidemiology study by Court-Brown et al. [1] and Hemmann et al. [3] due to a different grouping of fracture locations and the presentation of data, respectively. Other studies tend to focus on hip, pelvis, and vertebrae fractures while grouping other fractures into body regions, such as the "lower leg" and "upper arm" [1].

Despite only commenting on different locations of tibial fractures, the study by Wennergren et al. agrees with our observation that the tibial shaft had the highest incidence of open fractures out of all the tibial locations [5]. Despite not commenting on the epidemiology and grouping of lower limb fractures by whole bones, a 2020 audit on open lower limb fractures in the UK trauma system highlighted the prevalence of open fractures in the lower limb. The study found that tibia fractures were most likely, followed by the fibula, femur, fore, and hindfoot fractures [12]. It would be interesting to see further breakdown of these data to see if more precise locational analysis agrees with the observations made in our study.

Furthermore, another factor that affects limb fractures is obesity. Obesity is a growing problem in the UK, with the proportion of the population who are classified as obese rising from 14.9% in 1993 to 28.0% in 2021 [13]. This rise is worrying due to the significantly increased risk that obesity contributes to mortality and morbidity, such as cardiovascular disease, cancer, diabetes, and liver disease [14]. High BMI was previously considered protective against fractures due to the improved bone mineral density from increased weight [15]; however, more recent studies have shown that it is a protective factor and the relationship between obesity and fractures is more complex than initially thought and reliant on gender. A recent study by Turcotte et al. revealed that women with a BMI between 27.5 and 40 had an increased risk of distal lower limb fractures compared with women with a BMI of 25. However, in men, there was no observed relationship between fracture risk and BMI [16]. Court-Brown et al. also performed a study that correlated the BMI and fracture location [15]. In males and females, ankle fractures were associated with an increased BMI. However, there was a negative relationship between the proximal femur and BMI. Despite the observations and relationships made, the paper by Court-Brown et al. concludes that there is no link between fractures and BMI due to the many factors likely to be involved in fracture epidemiology [15].

The surgical management of fractures included external fixation, intramedullary nail, or plate fixation. In general, femur fractures were more often surgically managed than tibial fractures, with the highest rate of 92% occurring for the femoral shaft. Intramedullary nail fixation is the gold standard for femoral shaft fracture management in high-income countries, as incorrect treatment can lead to limb shortening and deformities [1]. Meanwhile, for tibial fractures, nonoperative management of the tibia has a high success rate if the aliment of the fracture is maintained. Surgery for tibial fractures only occurs if the fracture is associated with significant soft tissue injury [17-19]. Lateral malleolus fractures were most often conservatively managed, with just 77% requiring surgery. Our study did not look into the change in surgical management for each fracture location over time; however, Beerekamp et al. observed that there is a rising trend to treat upper limb fractures surgically, which has also been observed elsewhere, and a decreasing trend in surgical managed lower limb fractures [4].

Fractures of the lateral malleolus also required the shortest average hospital admission at 9.9 days, with femur subtrochanteric fractures requiring the longest at 21.6 days. The average time for admission to discharge was 14.6 days. Identifying the average length of hospital stay associated with fracture location enables hospitals to manage their resources and patients more effectively. It also helps the development of "clinical pathways" for inpatient treatment. Furthermore, analyzing the size of the hospital could elucidate operational bottlenecks and areas for improvement. This analysis frees up more hospital beds, decreases the risk of hospital-acquired infection and medical side effects, and can improve the quality of the patient's treatment [20].

Very little literature associates fracture location and ASA grade; however, one reason for peri-trochanteric fractures having a higher ASA grade could be the higher mean age related to these fractures, as discussed below. Interestingly, the literature has identified ASA grade as an independent predictor of readmission after a fracture [21,22].

Each fracture location, alongside the entire cohort, was split into each gender and had their total frequencies graphed with respect to age. In our study, subtrochanteric and peri-trochanteric fractures were subsequently grouped as "trochanter" fractures, and distal tibia, medial malleolus, and lateral malleolus were grouped as "ankle" fractures. Each fracture location had all fracture incidences graphed by age group in intervals of five years and separated by gender. This method was also used in the most recent paper investigating fracture epidemiology in the UK by Court-Brown et al. [1].

According to the literature, there is a rise in fracture incidence for females with age, which reflects the estrogen deficiency that occurs in menopausal and post-menopausal increasing fracture risk [23]. For males, the peak incidence is around the third decade of life, with the decline afterward being slow. This initial peak, observed in young males, reflects that men take higher risks and are more frequently more likely to suffer high-energy trauma [24].

Another critical factor in the epidemiology of adult lower limb fractures is the association with underlying conditions such as osteoporosis. A study conducted in the UK by Kaye et al. found that diagnosed osteoporosis was significantly associated with the risk of lower limb fractures [2]. This suggests that efforts to prevent and manage osteoporosis could dramatically reduce the incidence of lower limb fractures in adults. The established link between age and fracture incidence is a significant observation in an aging population such as the UK. In the coming years, the number of fractures due to osteoporosis will likely increase and make up a substantial proportion of healthcare spending. Thus, identifying osteoporotic fractures will have implications for the prevention, detection, treatment, and management of these osteoporotic fractures. Fractures traditionally considered osteoporotic were fractures of the proximal femur, thoracolumbar vertebrae, distal radius, and proximal humerus [1]. However, recently there have been suggestions that femoral fractures other than the proximal part and shaft fractures of the tibia and fibula in women were osteoporotic [1].

Our study graphed the incidence of fractures overall and each fracture location over time, both by year and by quarter. A German study looked at the changes in fracture location incidence over time in each age group [3]. In contrast, our study looked at the difference in fraction incidence over time with all the age groups combined. Regarding the timing of injuries, our study ran from 2015 to 2021. When each fracture location is graphed separately, there is no dramatic drop in one specific fracture location in the second quarter of 2020. The overall cohort decrease could be explained by the first COVID lockdown that happened in the UK between 23 March 2020 and 23 June 2020 [25]. This variation of fracture incidence could be driven by summer months, possibly explained by increased recreational activities due to warmer weather [11]. Wennergren et al. investigated the seasonal variation in tibial fractures across the year and graphed them according to the mechanism of the injury. They observed an increase in fractures in the winter months, which was caused by simple falls, possibly due to the colder weather. However, in the summer months, traffic accidents were the most common mechanism of injury, possibly due to people travelling more [5].

Limitations 

Our study had limitations within which our findings must be interpreted. As previously mentioned, our overall incidence of lower limb fractures was lower than quoted in other studies [1,2]. One of the main reasons for this could be that we are comparing against incidence values that consider fracture locations other than the lower limb [1]. Furthermore, the defined population that the trauma centre serves is uncertain, as ambulance dispatch and people visiting hospital services who are closer to them will influence the overall incidence of the fractures that we reported in this study. This uncertainty in the incidence estimate could be minimised by carrying out a whole nation study on lower limb fractures, similar to the method seen in the study in Germany [3]. However, carrying out and coordinating a national study collecting data across many trusts on lower limb fractures would be very challenging but would have the advantage of revealing differences in service needs for fracture management across the UK.

A factor that could have contributed to our study's low reported fracture incidence could be that, if multiple parts of the same bone were fractures, it was recorded as a single fracture. For example, if a patient had a communicated proximal humerus fracture, our methods would consider this a single proximal humeral fracture. Most orthopaedic research papers use the AO/OTA fracture classification, which is often used in research. However, we used the ICD-10 coding at the time of admission. Most other epidemiology papers also use the ICD-10 coding, with Wennergren et al.'s paper being the exception [5]. Using the ICD-10 coding may not be a limitation as our paper aims to provide policymakers with information about the population's epidemiology and needs, not to perform further research into the outcome of specific fracture types.

Our study did not consider any patient comorbidity data, which could have revealed comorbidities that could act as potential risk factors for specific fractures. Despite only using data from GP practices, the first paper investigating the epidemiology of lower limb fractures in the UK collected comorbidity and fracture data. They found that dementia accounted for the highest attributable risk for a femur fracture in people aged 80 years or older. In contrast, smoking was the attributable risk factor for the 50-79-year-old age group. This paper by Kaye et al. also collected data on the mechanism of injury, which our study could not do. Road collisions were identified as the highest relative risk of lower limb fracture [2]. Future studies should investigate lower limb fracture locations and the relationship to mechanisms of injury and patient comorbidities to characterize the epidemiology of these fractures further and identify risk factors to inform prevention on a multifactorial basis.

As well as the suggestions made above, future research directions could be a systematic or scoping review investigating the fracture incidence across various countries for specific lower limb fracture locations. This could reveal other contributing factors to fracture rates such as the climate, healthcare structure, and socioeconomic status of that country.

## Conclusions

There have been other studies defining the epidemiology of lower limb fractures being undertaken in other countries. However, to our knowledge, this paper is the first study of this type in the UK since 2006. This study aimed to define the epidemiological patterns in the fracture data for the lower limb, helping reveal demographic associations and relative incidences. This information will inform both clinicians and policy-makers on the needs of the population, help tailor health services to the population, and provide information to map the different healthcare needs over time, as well as between different countries.
